# Prenatal *p,p´*-DDE Exposure and Neurodevelopment among Children 3.5–5 Years of Age

**DOI:** 10.1289/ehp.1205034

**Published:** 2012-11-13

**Authors:** Luisa Torres-Sánchez, Lourdes Schnaas, Stephen J. Rothenberg, Mariano E. Cebrián, Erika Osorio-Valencia, María del Carmen Hernández, Rosa María García-Hernández, Lizbeth López-Carrillo

**Affiliations:** 1 National Institute of Public Health, Morelos, Mexico; 2 National Institute of Perinatology, Mexico City, Mexico; 3 Department of Toxicology, CINVESTAV, Mexico City, Mexico

**Keywords:** McCarthy scale, Mexico, neurodevelopment, organochlorines compounds, prenatal exposure, prospective cohort

## Abstract

Background: The results of previous studies suggest that prenatal exposure to bis[*p*-chlorophenyl]-1,1,1-trichloroethane (DDT) and to its main metabolite, 2,2-bis(*p*-chlorophenyl)-1,1-dichloroethylene (DDE), impairs psychomotor development during the first year of life. However, information about the persistence of this association at later ages is limited.

Objectives: We assessed the association of prenatal DDE exposure with child neurodevelopment at 42–60 months of age.

Methods: Since 2001 we have been monitoring the neurodevelopment in children who were recruited at birth into a perinatal cohort exposed to DDT, in the state of Morelos, Mexico. We report McCarthy Scales of Children’s Abilities for 203 children at 42, 48, 54, and 60 months of age. Maternal DDE serum levels were available for at least one trimester of pregnancy. The Home Observation for Measurement of the Environment scale and other covariables of interest were also available.

Results: After adjustment, a doubling of DDE during the third trimester of pregnancy was associated with statistically significant reductions of –1.37, –0.88, –0.84, and –0.80 points in the general cognitive index, quantitative, verbal, and memory components respectively. The association between prenatal DDE and the quantitative component was weaker at 42 months than at older ages. No significant statistical interactions with sex or breastfeeding were observed.

Conclusions: These findings support the hypothesis that prenatal DDE impairs early child neurodevelopment; the potential for adverse effects on development should be considered when using DDT for malaria control.

Recent epidemiological studies suggest that prenatal exposure to bis[*p*-chlorophenyl]- 1,1,1-trichloroethane (DDT), and its main metabolite 2,2-bis(*p*-chlorophenyl)-1,1-dichloroethylene (DDE), impairs psychomotor development (PD) during the first year of life (Eskenazi et al. 2006; Ribas-Fitó et al. 2003; Torres-Sánchez et al. 2007). However, information about the persistence of this association during early childhood is scarce and contradictory.

Studies using the Bayley Scale (Bayley 1993) to assess child neurodevelopment after 12 months of age have reported that the negative association between prenatal DDE exposure and PD during the first year of life is not evident at 24 (Eskenazi et al. 2006) or 30 months of age (Torres-Sánchez et al. 2009). Only three cohort studies have published results for children at ≥ 4 years of age. In 1991, Gladen and Rogan (1991) applied McCarthy’s Scales of Children’s Abilities to 712 children at 3, 4, and 5 years of age, and found no association between transplacental DDE exposure (median in maternal serum = 12.6 ng/mL) and child neurodevelopment. Similarly, Ribas-Fitó et al. (2006) assessed child neurodevelopment at 4 years of age in 475 children from two cohorts in Spain with a median umbilical cord serum DDE level of ~ 0.94 ng/mL and found no statistically significant associations with DDE; however compared with serum DDT < 0.05 ng/mL, serum DDT > 0.20 ng/mL was associated with statistically significant decreases of –5.9 points in the general cognitive index (GCI), and of –7.9 and –10.9 points in the verbal and memory components, respectively, of McCarthy’s Scales (McCarthy 1972). In the third study, the Conners’ Rating Scale for Teachers (Conners 1997) was applied to 607 children 7–11 years of age residing in Massachusetts; children in the highest versus lowest quartile of umbilical cord concentrations (overall DDE median = 0.31 ng/g) were almost twice as likely to be classified as having attention deficit hyperactivity disorders like behaviors (Sagiv et al. 2010).

In 2001 we started a perinatal cohort study in four municipalities in the state of Morelos, Mexico, an endemic area for malaria where DDT was used until 1998 as part of an anti-malaria campaign. DDE serum medians in 394 mothers during pregnancy were 7.7 ng/mL (wet weight) and 1020.4 ng/g lipid. A doubling of serum DDE during the first trimester of pregnancy was associated with a 0.52-point reduction in the Bayley Psychomotor Development Index from 1 to 12 months of age (Torres-Sánchez et al. 2007). In a cross sectional evaluation, at 1 month ± 7 days (Bahena-Medina et al. 2011) and in a longitudinal assessment from 12 to 30 months of age (Torres-Sánchez et al. 2009), no associations were observed. This report includes additional analyses of DDE and neurodevelopmental outcomes (based on McCarthy’s Scales) at 42, 48, 54, and 60 months of age.

## Methods

From 1 January 2001 through 30 June 2005, a prospective perinatal cohort study was assembled in Morelos, Mexico. Women were invited to participate during the prenuptial talks required by law for the formalization of civil marriage. Eligibility criteria included no history of chronic diseases (thyroid, heart, liver, kidney, or gastrointestinal disorders), not being treated with anticonvulsant drugs, and not breastfeeding at the time of the baseline interview. Those who agreed to participate in the study were interviewed before they became pregnant to obtain information on sociodemographic characteristics, diet, and reproductive history, and were followed up until they became pregnant. During their pregnancy they were monitored in relation to their pregnancy evolution (including maternal anthropometry) and dietary information, and blood samples were drawn in each trimester to measure DDE levels. Each participant signed an informed consent letter. The study was approved by the National Institute of Public Health’s Ethics Committee. Details concerning the cohort-assembling procedures have been published elsewhere (Torres-Sánchez et al. 2007).

**Figure 1:**
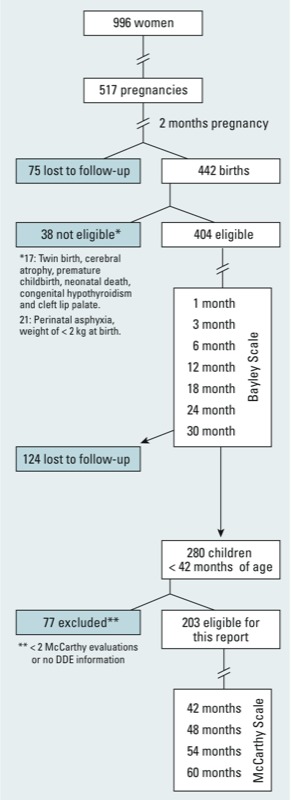
Morelos Perinatal Cohort Study 2001–2009.

During 8.5 years of follow-up, 442 live first births occurred among the women (80% were primiparous) who had enrolled before their pregnancy (Figure 1). Eligibility criteria for this analysis were at least two assessments in the child for neurodevelopment between 42 and 60 months of age, birth weight of ≥ 2 kg, no history of complicated birth, and available maternal DDE serum levels for at least one trimester of pregnancy. There were 203 eligible children. Characteristics of 124 children who were lost to follow-up before 42 months of age and of 77 children who were excluded (noneligible) are shown in Table 1. The main reasons for loss to follow-up were change of address and lack of parental interest.

**Table 1:** Parental, infant, and selected family characteristics of the study sample.

Characteristic	Lost to follow-up before 42 months (n = 124)	Excluded (n = 77)	Included in the analysis (n = 203)
Parental
Maternal age (years)	22.2 ± 4.4	23.5 ± 4.6	21.8 ± 3.9*
Maternal education (years)	10.7 ± 3.2	11.8 ± 3.5	10.6 ± 3.1*
Maternal paid occupation	44.7	58.4	47.8
Maternal civil statusa married	—	88.0	93.1
Maternal intelligence quotient	88.3 ± 13.2	91.6 ± 14.6	87.1 ± 11.2*
Paternal education (years)	11.4 ± 3.2	11.0 ± 3.5	10.8 ± 3.2
Infant
Male	51.7	51.9	57.6
Birth weight (kg)	3.2 ± 0.4	3.2 ± 0.4	3.2 ± 0.4
Height at birth (cm)	50.4 ± 2.5	50.2 ± 2.5	50.3 ± 2.4
Gestational age (week)	39.2 ± 1.6	38.8 ± 1.3	39.3 ± 1.4
Cesarean birth	60.0	68.8	55.7
Breastfeeding
Never	—	7.8	7.4
≤ 12 weeks	—	25.9	19.7
> 12 weeks	—	66.2	72.9
School attendancea	—	15.4	35.5
Family
Nuclear familyb	50.8	55.8	53.7
HOME scale < 25c	—	14.5	12.4
Values are percent or mean ± SD. aAt 48 months evaluation. bLiving with first-degree relatives only. cHome environments considered to be of low quality. *ANOVA test, included vs. excluded p < 0.05.			

*Postnatal follow-up*. Following their birth, the children were assessed at 1, 3, 6, 12, 18, 24, 30, 36, 42, 48, 54, and 60 months of age. During the first visit at 1 month of age, information was obtained about characteristics related to the child’s birth and the beginning of breastfeeding. In each subsequent visit, a structured in-person interview was administered to obtain information about the type and duration of breastfeeding, the child’s dietary habits and health status, and the place where the child was looked after (home or child care center). In addition, the children were weighed and measured for length and head circumference, and their neurodevelopment was assessed. Child neurodevelopment tests varied according to the child’s age, with the Bayley Scale (Bayley 1993) used before 42 months of age, and the McCarthy Scales of Children’s Abilities (McCarthy 1972) used ≥ 42 months of age.

*McCarthy Scales of Children’s Abilities*. The McCarthy Scales of Children’s Abilities assesses cognitive and motor skills through five scales: perceptual-performance, quantitative, verbal, memory, and motor skills. The perceptual-performance, quantitative, and verbal scales are summed to derive a GCI that is considered equivalent to the intellectual coefficient provided by other tests (Goldstein and Hersen 1984). We used a Spanish-language McCarthy Scales–validated version that has been used in Spain (McCarthy 2006) as well as the U.S. McCarthy tables (McCarthy 1972) to normalize our McCarthy Scales results to a specific age in months. The test was administered by three psychologists who had no knowledge of the DDE prenatal concentrations. Interobserver reproducibility was 0.99.

*Determination of DDT and DDE.* Blood samples (7 mL) were obtained during the baseline interview and/or at each trimester visit. After centrifugation, the serum obtained was stored at –70°C in glass vials (prewashed with purified hexane) covered with a Teflon cap, until analyzed.

The levels of DDE and DDT in serum were determined by means of gas chromatography with an electron capture detector (model 3400; Varian, Inc., Palo Alto, CA, USA), following the protocol recommended by the U.S. Environmental Protection Agency (1980). Concentrations of DDE and DDT were determined on a wet basis as nanograms per milliliter (parts per billion), and on a lipid basis as nanograms per gram of lipid. The detection limit was 0.05 ng/mL and 0.0045 ng/mL for DDE and DDT, respectively. All serum samples were above the limit of detection for DDE, whereas an average of 24.1% (depending on the trimester of pregnancy) was above the limit of detection for DDT. Due to the high proportion of samples < LOD for DDT, we report only the association with DDE.

For internal quality control, each of the serum samples was fortified with aldrin and the average recovery was 98.15 ± 8.8%. For every 10 study samples, one sample of bovine serum with known quantities of β-hexachlorocyclohexane (β-HCH), aldrin, hexachlorobenzene (HCB), DDE, and 1,1-dichloro-2,2-bis(*p*-chorophenyl) ethane (*p,p´*-DDD) was analyzed, with recovery of 100.8, 100.01, 100.91, 103.4, and 104.1%, respectively. Additionally, one randomly selected sample was analyzed in duplicate in each batch, with a coefficient of variation of 4.4% and 8.3% for DDE and DDT, respectively. The results of the external quality control comparing DDE levels measured in 10 split serum samples by our laboratory (CINVESTAV) and M. Wolff’s laboratory in the Division of Environmental and Occupational Medicine at Mount Sinai Medical School showed a coefficient of Bland–Altman correlation of 0.98.

*Key covariables.* As part of the study, the IQ of each mother was measured by means of the Spanish version of Wechsler’s Adult Intelligence Scale (Wechsler 1981). The quality of home stimulation was assessed through total score in the HOME (Home Observation for Measurement of the Environment) scale (Caldwell and Bradley 1984). In addition, blood lead levels were determined in a subsample of women. Duplicate analysis of blood lead levels was performed in ESA laboratories (Environmental Science Associates Laboratories, Inc., Chelmsford, MA, USA), using a voltammetric anodic separation method. Samples with mean levels < 5 μg/dL were reanalyzed using atomic absorption spectrometry (model 3000; Perkin-Elmer Inc., Norwalk, CT, USA). External quality control samples were provided by Centers for Disease Control and Prevention laboratories (Atlanta, GA, USA) and Pennsylvania State Blood Lead Proficiency Testing Program (Exton, PA, USA).

*Statistical analysis.* Included children were compared to excluded children and those who were lost to follow up before 42 months of age, according to selected maternal and child characteristics by analysis of variance (ANOVA) or chi-square test as appropriate.

DDE serum concentrations during each trimester of pregnancy were right-skewed, and were natural logarithm transformed, and Spearman correlations among them across trimesters were calculated. As an indicator of timeliness of DDT exposure, we calculated DDT/DDE ratio dividing DDT on DDE serum concentrations; ratios < 1 suggest a nonrecent DDT exposure. Using mixed-effects models, we estimated the mean of each McCarthy component at 42, 48, 54, and 60 months. This last variable was further coded as 1, 2, 3, or 4 to test for trend.

The association between prenatal DDE exposure and child neurodevelopment during 42–60 months of age was estimated through mixed effect multivariate models, for each component of the McCarthy Scale (perceptual-performance, quantitative, verbal, memory, and motor) and separately for each trimester of pregnancy. The models’ general equation is the following:

*Y_ij_* = *DDE_i_*β + *X_i_*β + *Z_ij_*γ*_i_* + ε*_ij_*, [1]

where *Y_ij_* corresponds to a McCarthy component (i.e., perceptual-performance, quantitative, etc.) in participant *i* and time *j* (where *j* = 42, 48, 54, or 60 months of age), *DDE_i_* is the natural logarithm of DDE serum concentrations during each trimester or its average during pregnancy. Potential adjustment factors with fixed effect (*X_i_*) were added one at a time to a crude model: maternal age and education (years), occupation (paid/unpaid), parity (first pregnancy/more than one pregnancy), maternal IQ, mother’s marital status at child’s age of 4 years (in a stable union yes/no), father’s education (finished years), gestational age at birth (weeks of pregnancy), type of birth (vaginal/cesarean), child’s sex (female/male), birth weight (kilograms), breastfeeding history (none, ≤ 12 weeks, and > 12 weeks), attendance at a child care center at 4 years of age (yes/no), HOME scale results at 6 months of age, and blood lead (micrograms per deciliter, in a subsample of the study population). Only those that changed the crude estimator of interest > 10% remained in the final model. Finally, *Z_ij_*γ*_i_* are random effects variables for age and anthropometry for assessment *j*. A linear random slope of age at evaluation and an unstructured covariance were considered for all models. To check the sensitivity of our estimates to the influence of missing maternal DDE values in any trimester of pregnancy, we repeated the analyses in the subset of children (*n* = 84) with two or more neurological evaluations, but whose mother had DDE values in all trimester of pregnancy.

To facilitate the interpretation, the (beta) regression coefficient corresponding to the logarithmic levels of DDE in each trimester of pregnancy was multiplied by 0.69 (lnDDE/log_2_DDE), to estimate the change in each McCarthy Scales component for a doubling of the serum DDE level.

To assess potential modification of the relationship between prenatal DDE exposure and McCarthy Scale components by the child’s age at assessment, sex, or breastfeeding history (yes/no), we added product interaction terms between DDE (continuous) and each potential modifier to final models (one at a time). Interaction term *p*-values < 0.10 were considered to be statistically significant.

The normality of the residuals in final models was assessed using the Shapiro–Wilks test, histograms, and quartile graphs. Model predictions were graphed against standardized residuals to assess heteroscedasticity. For all evaluations, *p* < 0.05 was considered significant. All analyses were carried out using STATA software version 12.1 (StataCorp, College Station, TX, USA).

## Results

Males were more prevalent than females in the study population included in this analysis (Table 1). About one-half were born by cesarean section, consistent with the high rate of cesarean births reported for Mexico (Villar et al. 2006). The average birth weight was 3.2 kg and the average length was 50.1 cm. Most of the children (72.9%) were breastfed during at least the first 12 weeks, and at 4 years of age at least 35.5% had attended a child care center. Roughly one-half the mothers had a paid job, and most of them were in a stable union at the time of the 48-month evaluation, with an average IQ of 87.1. Only 12.4% of children received poor stimulation at home (HOME score ≤ 25) In general, participants were similar to nonparticipants regarding parental, infant, and family characteristics; however, differences in maternal age, education, and IQ were statistically significant, with slightly higher values in excluded children compared with participating children (Table 1). Correlation coefficients between serum DDE levels measured during different trimesters indicated no statistically significant variation through the pregnancy (Table 2). Median serum DDE levels varied between 7.6 ng/mL in the first trimester (1255.39 ng/g lipid) to 8.9 ng/mL in the third trimester (812.7 ng/g lipid). The DDT/DDE ratio showed that the study population was not recently exposed to DDT (Table 2). These patterns did not differ noticeably between the full sample and the subset of 84 women who had serum from all trimesters (data not shown).

**Table 2:** Maternal DDT and DDE concentrations during pregnancy.

Compound	Wet basis (ng/mL)a			Lipid basis (ng/g)b		
	P10	Median	P90	P10	Median	P90
DDEc
1st trimester (n = 176)	1.84	7.64	23.05	259.26	1255.40	4964.21
2nd trimester (n = 123)	1.32	8.22	23.41	154.39	1138.16	2857.14
3rd trimester (n = 144)	1.7	8.95	29.20	153.23	812.75	2919.00
DDTc
1st trimester (n = 176)	0.0045	0.0045	0.0159	0.0123	0.0123	31.48
2nd trimester (n = 123)	0.0045	0.0045	0.03	0.0123	0.0123	2.54
3rd trimester (n = 144)	0.0045	0.0045	0.03	0.0123	0.0123	4.21
DDT/DDE ratio
1st trimester (n = 176)	0.0003	0.001	0.007	4.68–6	1.72–5	0.023
2nd trimester (n = 123)	0.0002	0.001	0.004	4.47–6	1.61–5	0.002
3rd trimester (n = 144)	0.0002	0.001	0.006	5.21–6	2.29–5	0.007
P10 and P90 are 10th and 90th percentiles. aSpearman’s r: 1st vs. 2nd trimester = 0.58; 1st vs. 3rd trimester = 0.47; 2nd vs. 3rd trimester = 0. 67; p < 0.05 (n = 84). bSpearman’s r: 1st vs. 2nd trimester = 0.59; 1st vs. 3rd trimester = 0.53; 2nd vs. 3rd trimester = 0.65; p < 0.05 (n = 84). cDDE: all samples were above detection limit; DDT: on average 24.1% samples were above detection limit.							

Results in the McCarthy Scale for all children at each measurement were found to be within the normal limits for each component of the test. In most cases, girls showed higher performance on scale components than boys, with significantly higher scores for perceptual-performance at 42 months and for the GCI, verbal, and memory components at 42 and 48 months. GCI, perceptual-performance, verbal, and motor components showed a statistically significant increase with increasing age among boys, but not girls (Table 3).

**Table 3:** Mean scores (± SD) of McCarthy Scales of Children’s Abilities components by age at evaluation and sex of child.

McCarthy component	Male					Female				
	Age at evaluation (months)				p for trend	Age at evaluation (months)				p for trend
	42 (n = 82)	48 (n = 98)	54 (n = 103)	60 (n = 94)		42 (n = 55)	48 (n = 71)	54 (n = 74)	60 (n = 69)	
GCI	93.5 ± 14.4a	91.9 ± 15.1a	96.1 ± 13.9	96.2 ± 14.2	0.000	99.9 ± 14.1	96.7 ± 13.8	96.6 ± 11.7	96.4 ± 11.1	0.13
Perceptual-performance	48.1 ± 9.0a	49.3 ± 9.6	50.4 ± 7.7	50.7 ± 9.0	0.005	52.8 ± 8.8	50.9 ± 8.4	50.8 ± 7.4	50.5 ± 8.1	0.47
Quantitative	42.8 ± 7.9	41.4 ± 10.2	41.1 ± 10.7	42.6 ± 9.8	0.83	42.7 ± 8.5	42.2 ± 9.6	42.3 ± 9.4	41.5 ± 8.7	0.11
Verbal	46.5 ± 8.8a	45.6 ± 8.5a	48.5 ± 9.1	48.4 ± 8.9	0.000	50.6 ± 9.4	48.8 ± 8.6	47.9 ± 8.7	48.6 ± 6.7	0.10
Memory	46.9 ± 7.7a	46.0 ± 8.0a	46.9 ± 8.4	46.5 ± 8.9	0.87	49.7 ± 7.9	48.8 ± 7.6	47.2 ± 8.7	47.4 ± 8.1	0.72
Motor	47.6 ± 9.9	48.2 ± 9.7	50.2 ± 9.4	50.7 ± 10.2	0.02	49.9 ± 8.6	47.2 ± 8.5	50.0 ± 7.3	49.9 ± 9.2	0.70
aMale vs. female p < 0.05.											

A doubling of serum DDE levels (nanograms per milliliter) in the second and third trimesters was negatively associated with GCI, quantitative, verbal, and memory component scores, though associations were significant for the third trimester only (β = –1.37, β = –0.88, β = –0.84, and β = –0.80, respectively) (Table 4). DDE was negatively associated with quantitative and memory components throughout pregnancy among the 84 children whose mothers had DDE measurements for all three trimesters, and associations with DDE in the third trimester were statistically significant and higher than estimates based on all children for GCI, quantitative, verbal, and memory component scores (β = –2.01, β = –2.06, β = –1.14 and β = –1.26, respectively). The association with average DDE levels during pregnancy among children whose mothers had DDE measurements for all trimesters was significant for the quantitative component only (β = –1.78; 95% CI: –3.2, –0.4; *p* = 0.01). Estimated associations were similar when natural log-transformed DDE was modeled on a lipid-weight basis and when lipid concentration was included as an independent covariable. Blood lead levels, which were available for 84, 77, and 97 mothers in the first, second, and third trimester (geometric means of 5.7, 5.0, and 5.9 μg/dL, respectively), did not confound associations between developmental scores and DDE (data not shown).

**Table 4:** Association between prenatal DDE exposure and McCarthy components among children [β (95% CI)].

McCarthy component	Prenatal DDE levels (ng/mL)						
	1st trimester (n = 176)a	2nd trimester (n = 123)a	3rd trimester (n = 144)a	1st trimester (n = 84)b	2nd trimester (n = 84)b	3rd trimester (n = 84)b	Average DDE exposure (n = 84)b
GCIc	0.74 (–0.37, 1.86)	–0.10 (–1.32, 1.13)	–1.37 (–2.56, –0.19)**	0.73 (–1.11, 2.59)	–0.03 (–1.60, 1.54)	–2.01 (–3.64, –0.38)**	–0.72 (–2.75, 1.30)
Perceptual-performanced	0.46 (–0.24, 1.14)	0.28 (–0.60, 1.14)	–0.41 (–1.18, 0.36)	0.51 (–0.69, 1.72)	0.45 (–0.59, 1.51)	–1.02 (–2.10, 0.05)	–0.07 (–1.43, 1.29)
Quantitativee	0.44 (–0.32, 1.20)	–0.32 (–1.17, 0.51)	–0.88 (–1.67, –0.10)**	–0.74 (–2.02, 0.54)	–0.63 (–1.77, 0.50)	–2.06 (–3.15, –0.95)**	–1.78 (–3.20, –0.36)**
Verbalf	0.17 (–0.54, 0.87)	–0.19 (–0.97, 0.60)	–0.84 (–1.63, –0.56)**	0.34 (–0.74, 1.43)	–0.21 (–1.17, 0.75)	–1.14 (–2.17, –0.11)**	–0.46 (–1.72, 0.79)
Memoryg	0.027 (–0.64, 0.70)	–0.28 (–1.02, 0.48)	–0.80 (–1.52, –0.08)**	–0.29 (–1.37, 0.80)	–0.51 (–1.43, 0.41)	–1.26 (–2.20, –0.32)**	–1.06 (–2.25, 0.13)
Motorh	0.41 (–0.33, 1.17)	0.26 (–0.59, 1.10)	–0.03 (–0.87, 0.80)	1.06 (–0.14, 2.27)	0.70 (–0.32, 1.74)	–0.10 (–1.19, 0.99)	0.77 (–0.55, 2.10)
All models were adjusted for child’s age at examination, mother’s education and marital status when child was 4 years old, and HOME score. β, change in score associated with a doubling of prenatal maternal serum DDE levels (ng/mL). aChildren with two or more neurological evaluations and maternal DDE levels in at least one trimester of pregnancy. bChildren whose mothers had DDE measurements for all three trimesters. cAdditionally adjusted for sex of child and attendance at a child care center. dAdditionally adjusted for sex of child, height at birth and weight at the time of the evaluation, and attendance at a child care center. eAdditionally adjusted for maternal IQ and child’s birth weight. fAdditionally adjusted for maternal age and IQ, birth weight, infant height at the time of the evaluation, and attendance at a child care center. gAdditionally adjusted for maternal age and IQ, birth weight, and attendance at a child care center. hAdditionally adjusted for gestational age, type of childbirth, child’s weight and height at the time of the evaluation, and attendance at a child care center. **p < 0.05.							

**Figure 2:**
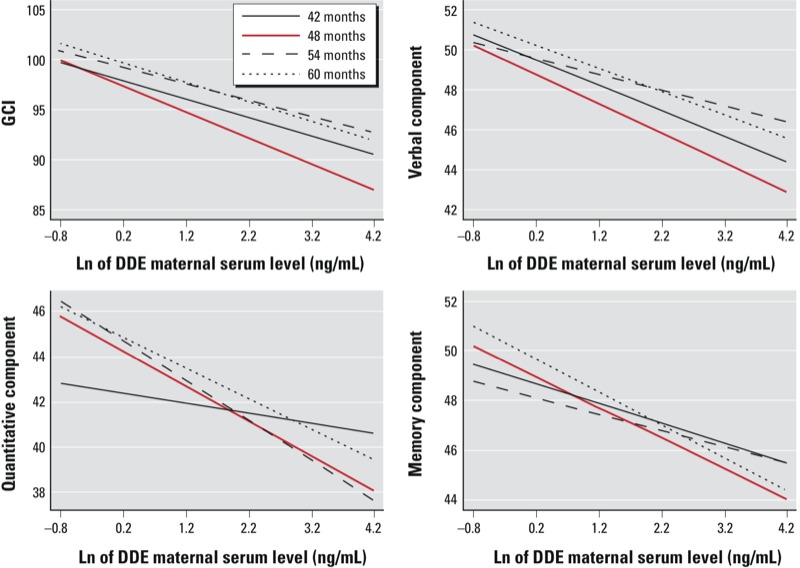
Maternal serum DDE during the third trimester and McCarthy Scales of Children’s Ability components according to age at evaluation. Interactions between serum DDE (continuous) and age at examination for each McCarthy component estimated through mixed-effects multivariate models. All models were adjusted for child’s age at examination, mother’s education and marital status when child was 4 years old, and HOME score. Additionally, the GCI model was adjusted by sex of child and attendance at a child care center; the quantitative model also was adjusted by maternal IQ and child’s birth weight; the verbal model also was adjusted by maternal age and IQ, birth weight, infant height at the time of the evaluation, and attendance at a child care center; the memory model also included maternal age and IQ, birth weight, and attendance at a child care center.

The association between maternal serum DDE during the third trimester and the quantitative component score varied depending on age at assessment, with a statistically significant interaction at 54 months, compared to 42 months, resulting in a weaker association at 42 months (Figure 2). No significant interactions between other components and age (Figure 2), or between any components and sex or breastfeeding (data not shown), were observed (interaction *p*-values > 0.10). The HOME scale was a statistically significant positive predictor of all McCarthy test components (data not shown).

## Discussion

Findings from this study suggest that DDE prenatal exposure has a long-term effect on child neurodevelopment. We observed that a doubling of serum DDE (nanograms per milliliter) during the third trimester of pregnancy was associated with a reduction of –1.37, –0.88, –0.84, and –0.80 points in the GCI, quantitative, verbal, and memory components of McCarthy scale at 3.5–5 years of age.

To date, only two additional studies have published results regarding prenatal DDE levels and neurodevelopment in preschoolers, with inconsistent results, probably due to methodological differences. Gladen and Rogan (1991) examined a prospective cohort in North Carolina (USA) with a larger sample size (up to 645) and higher maternal median DDE levels (12.6 ng/mL) than those in the present study. They only found a marginal negative reduction at 3, 4, and 5 years in quantitative skills. It is possible that the cross-sectional statistical approach that was used limited the power of that study to reach statistically significant associations, and/or residual confounding of key covariables (e.g., breastfeeding, socioeconomic level) influenced their findings. In Spain, Ribas-Fitó et al. (2006) performed a prospective cohort study of 475 children. They found a significant negative association between DDE in cord serum and the memory component of the McCarthy Scales at 4 years of age, and nonsignificant negative associations with the verbal component and GCI, consistent with our results. Median DDE levels were lower (0.9 ng/mL in umbilical cord blood) than in the present study population, and the mean quantitative component score in their population (18.34) was about two times lower than in our study sample. In addition, outcomes were tested only once, at about 4 years of age. It is worth noting that they found stronger associations with DDT.

In our cohort, maternal serum DDE level during the first trimester was negatively associated with psychomotor development (Bayley Scale) (Bayley 1993) during the first year of life (Torres-Sánchez et al. 2007), whereas maternal serum DDE during the third trimester was negatively associated with GCI, quantitative, verbal, and memory skills (McCarthy Scale) between 3.5 and 5 years of age. Although DDE exposure during pregnancy does not change substantially over a 9-month period, brain development is an ongoing process that comprises development, migration, and organization of neuronal cells through pregnancy. Future developmental functions may be affected differently by exposure at different developmental stages. Different tests have been designed to assess the development at different ages. The Bayley test was constructed under the premise that ability may or may not have been acquired by the testing age—a situation that may be related to the brain fetal development that occurs during the first trimester. Tests at older ages, such as the McCarthy Scales, may reflect dendritic ramifications formed during the latter part of pregnancy (Adams et al. 2000; Rice and Barone 2000; Volpe 2008).

The motor component of the McCarthy Scale was not associated with prenatal DDE exposure, consistent with a study of Mexican-American children evaluated using the Bayley Scale at 12 and 24 months of age (Eskenazi et al. 2006) and with a previous analysis of the present study cohort between 12 and 30 months of age (Torres-Sánchez et al. 2009), both of which suggest that observed associations between DDE and psychomotor development during the first year of life do not persist at later ages.

Thyroid hormone plays an important role in brain development through its effects on neuronal differentiation and maturation (Anderson et al. 2003), neuronal migration and proliferation (Lavado-Autric et al. 2003), and synaptogenesis and myelination (Porterfield 2000). A recent study of children followed up to 5.5 years of age showed that slight reductions in the resine triiodothyronine uptake ratio, an indication of the amount of thyroid-binding globulin sites unsaturated by thyroxine (T_4_), was negatively associated with child neuropsychological functions. In the same study, DDE exposure during pregnancy was also associated with reduced resine triiodothyronine uptake ratios in mothers and children (Julvez et al. 2011).

Experimental evidence suggests that DDE is an endocrine disruptor that may disturb thyroid hormone homeostasis. In rats, DDE was shown to lower free T_4_ levels, increase thyroid hormone receptors, reduce thyroid hormone–transporting protein, and increase thyroid hormone degradation through induction of hepatic enzymes (Liu et al. 2011). Another potential mechanism is that DDT may also affect neurodevelopment by reducing the density of muscarinic cholinergic receptors involved in neuronal transmission (Eriksson et al. 1992).

Interpretation of our results requires evaluation of potential alternative explanations. The associations observed among children with two or more neurological evaluations whose mothers had maternal DDE levels measured in all trimesters of their pregnancy were significant and more negative than associations estimated among children whose mothers had missing DDE measurements for the first and second trimesters. However, despite an attrition rate (up to 42 months) of about 30%, children were very similar in key parental, infant, and family characteristics independent of the number of missing DDE data values.

The high proportion of cesarean sections observed in this study is consistent with a regional survey that reported that Mexico has one of the highest proportions of deliveries by cesarean section in Latin America, with a range of 28–72% depending on the type of health service used (public, social security, private) (Villar et al. 2006). Therefore, we do not feel that the high proportion of cesarean sections should limit generalizability.

The potential for differential measurement error is low because the psychologists applying the McCarthy Scale had no knowledge of the prenatal exposure of each child. However, random error in the measurement of child neurodevelopment, exposure, or model covariates may have influenced results. In addition, the lack of validation of the McCarthy Scale in the Mexican population may reduce its sensitivity and specificity.

There are no published data regarding polychlorinated biphenyls, arsenic, or organophosphate levels in the study area, but there is evidence of lead exposure (Moline et al. 2000), so alternative models were adjusted by lead. The findings suggested that lead did not confound the observed associations (data not shown). However, the possibility of residual confounding should not be ruled out.

A limitation of our study was the lack of information regarding postnatal DDE exposure, which prevented us from assessing the role of this exposure in child neurodevelopment at 3.5–5 years of age. However, lack of effect modification by breastfeeding in the present study suggests that postnatal exposure from this source was not important. We also did not have information on postnatal lead exposure or infant nutrition. However, we believe that prenatal exposure is likely to be more influential than postnatal exposure for the development of the central nervous system.

## Conclusions

Our findings support the hypothesis that DDE prenatal exposure is negatively associated with child neurodevelopment at 3.5–5 years of age. Additional follow up is needed to determine if GCI, quantitative, verbal, and memory associations with prenatal DDE exposure is a key determinant of poor further academic performance, considering that DDT is still being used in some countries (Bouwman et al. 2011).
